# Biodegradable Tri-Block Copolymer Poly(lactic acid)-poly(ethylene glycol)-poly(l-lysine)(PLA-PEG-PLL) as a Non-Viral Vector to Enhance Gene Transfection

**DOI:** 10.3390/ijms12021371

**Published:** 2011-02-23

**Authors:** Chunhua Fu, Xiaoli Sun, Donghua Liu, Zhijing Chen, Zaijun Lu, Na Zhang

**Affiliations:** 1 School of Pharmaceutical Science, Shandong University, 44 Wenhua Xi Road, Ji’nan, 250012 Shandong Province, China; E-Mails: xiaolisun2008@yahoo.cn (X.S.); donghua0209@163.com (D.L.); czjn2001@yahoo.cn (Z.C.); 2 Department of Pharmacy, Shandong Medical College, 5460 Bicyclic Nan Road, Ji’nan, 250002 Shandong Province, China; E-Mail: Chunhua@163.com (C.F.); 3 School of Chemistry and Chemical Engineering, Shandong University, 27 Shanda Road, 250010 Ji’nan, China; E-Mail: z.lu@sdu.edu.cn (Z.L.)

**Keywords:** PLA-PEG-PLL, nanoparticles, non-viral gene vector, gene transfection, tri-block copolymer

## Abstract

Low cytotoxicity and high gene transfection efficiency are critical issues in designing current non-viral gene delivery vectors. The purpose of the present work was to synthesize the novel biodegradable poly (lactic acid)-poly(ethylene glycol)-poly(l-lysine) (PLA-PEG-PLL) copolymer, and explore its applicability and feasibility as a non-viral vector for gene transport. PLA-PEG-PLL was obtained by the ring-opening polymerization of Lys(Z)-NCA onto amine-terminated NH_2_-PEG-PLA, then acidolysis to remove benzyloxycarbonyl. The tri-block copolymer PLA-PEG-PLL combined the characters of cationic polymer PLL, PLA and PEG: the self-assembled nanoparticles (NPs) possessed a PEG loop structure to increase the stability, hydrophobic PLA segments as the core, and the primary ɛ-amine groups of lysine in PLL to electrostatically interact with negatively charged phosphate groups of DNA to deposit with the PLA core. The physicochemical properties (morphology, particle size and surface charge) and the biological properties (protection from nuclease degradation, plasma stability, *in vitro* cytotoxicity, and *in vitro* transfection ability in HeLa and HepG2 cells) of the gene-loaded PLA-PEG-PLL nanoparticles (PLA-PEG-PLL NPs) were evaluated, respectively. Agarose gel electrophoresis assay confirmed that the PLA-PEG-PLL NPs could condense DNA thoroughly and protect DNA from nuclease degradation. Initial experiments showed that PLA-PEG-PLL NPs/DNA complexes exhibited almost no toxicity and higher gene expression (up to 21.64% in HepG2 cells and 31.63% in HeLa cells) than PEI/DNA complexes (14.01% and 24.22%). These results revealed that the biodegradable tri-block copolymer PLA-PEG-PLL might be a very attractive candidate as a non-viral vector and might alleviate the drawbacks of the conventional cationic vectors/DNA complexes for gene delivery *in vivo*.

## 1. Introduction

Gene therapy, *i.e.*, the expression of genetic material with therapeutic activity in cells, holds great potation for the treatment of human diseases [1]. A gene delivery system, of either viral or non-viral vector, must be used to carry the foreign gene into the target cell. Despite the high transduction efficiency of viral vectors which are derived from viruses by the use of recombinant DNA techniques, their clinical potential should be fully understood in terms of issues related to production, safety and immune response that need to be addressed [2]. The safety concerns regarding the use of viral vectors in humans make non-viral delivery systems an attractive alternative. The non-viral vectors have recently gained increasing attention due to their stability, safety, ease of preparation, and are easily manufactured for large-scale production for treatment of numerous acquired or inherited human diseases [3,4]. Unfortunately, non-viral gene delivery vehicles often affect cell viability and have poor transfection efficiency.

The application of non-viral gene delivery vectors, including liposomes, cationic polymers and polymeric nanoparticles (NPs), could reduce or avoid immunogenicity and associated risks of toxicity. The versatility for formulation, sustained release properties, sub-cellular size and biocompatibility with tissues and cells make nanoparticles a promising system to achieve ideal gene tranfection [5]. The nanoparticles prepared by biocompatible and biodegradable poly (d,l-lactide-*co*-glycolide) (PLGA) and poly (d,l-lactide) (PLA) polymers have attracted much attention because of their favorable physicochemical characteristics in terms of safety, stability, the relative ease of large-scale production, and lack of intrinsic immunogenicity that make them suitable candidates for gene delivery application [6–8]. These biodegradable polymers undergo hydrolysis upon implantation into the body, forming biologically compatible and metabolizable moieties (lactic acid and glycolic acid) that are eventually removed from the body by the citric acid cycle [5]. However, an important problem with PLA is inadequate interaction between the polymers and cells, and these polymeric NPs can be easily displaced by serum proteins, which can lead to aggregation of NPs. Incorporation of additional excipients such as polyethylene glycol (PEG) has been attempted as a method to prevent the generation of an extremely acidic microenvironment inside the NPs on polymer degradation [9]. PEG has been used to coat the PLA NPs, and could often improve the solubility of the NPs, minimize their aggregation, reduce their interaction with proteins in the physiological fluid, and finally, produce a shielding effect that counteracts effective DNA complexation and steric stabilization to the gene vectors’ surfaces to improve transfection efficiency [10,11]. Because the PLA-PEG block copolymer has an amphiphilic character, it forms polymeric micelles in an aqueous milieu, the core is surrounded by a palisade of tethered PEG chains with an appreciably stretched conformation [12] that have predominant characteristics such as a long blood circulation time, biodistribution and lower interactions with the reticuloendothelial system (RES) [10]. Generally, plasmid DNA is encapsulated into PLA particles using the common water-in-oil-water (W/O/W) double emulsion/solvent evaporation method in order to protect plasmid DNA and control the release process [11,13]. However, these preparation methods can not guarantee the integrity and can even result in the degradation of DNA under the ultra-sonication or high speed homogenization which is necessary in the encapsulation process to obtain smaller particle sizes [14]. To protect the DNA cargo, one approach in the application of PLGA NPs for nucleic acid delivery uses adsorption of the anionic DNA molecules onto cationic NPs by the use of cationic surfactants, like cetyltrimethyl ammonium bromide (CTAB), and 3,2′-dimethyl-4-aminobiphenyl (DMAB) in the formulations [15]. In our previous studies, Zou *et al.* also investigated how gene loaded cationic PLA-PEG NPs modified by CTAB could successfully transfect a gene into HeLa cells even in the presence of serum [16]. However, there is no doubt that introduction of cationic surfactants would generate cytotoxicity. The polar and hydrophobic domains of cationic surfactants may have dramatic effects on both transfection and toxicity levels [17].

On the basis of PLA-PEG, as previously, in order to avoid the use of cationic surfactants, here we try to design a biodegradable tri-block copolymer poly(lactic acid)-poly(ethylene glycol)-poly(l-lysine) (PLA-PEG-PLL), which combines the characters of cationic polymer PLL, biodegradable polymer PLA and PEG, which was selected as the shell-forming segment due to its physicochemical characteristics, including high water solubility and significant chain mobility as well as its low toxicity [12]. The cationic polymer, poly(l-lysine) (PLL) has been widely applied in gene delivery vectors. The primary ɛ-amine groups of lysine in PLL could electrostatically interact with negatively charged DNA [18] and help to improve the affinity to proteins and cells. Based on the electrostatic binding with the tumor cell, PLL-based cationic and biodegradable polymeric micelles are expected to permeate tumor cells more feasibly [19]. In addition, due to the large number of active functional groups with amino, PLL could be modified with various kinds of ligands to achieve active targeting to tissues and cells.

Compared with various kinds of polymers, cationic polymer PLL was biodegradable but exhibited low transfection efficiency. PEI was considered as one of most efficient currently-available transfection reagents, but this molecule is not biodegradable and relatively cytotoxic. Cationic PLA-PEG NPs modified with cationic surfactants would induce cytotoxicity, while PLA-PEG-PLL NPs were biodegradable, induced low cytotoxicity and possessed a positive charge without any cationic surfactants. Based on these considerations, the main goal of the present work was to synthesize PLA-PEG-PLL and explore its applicability and feasibility as a non-viral vector for gene transport.

## 2. Results and Discussion

**Scheme 1.**
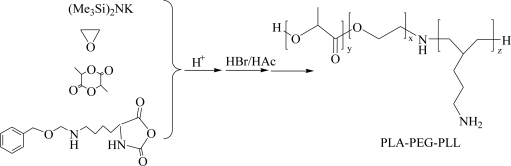
Schematic synthetic route of poly(lactic acid)-poly(ethylene glycol)-poly(l-lysine) (PLA-PEG-PLL).

The synthetic route of PLA-PEG-PLL is shown in Scheme 1. The method has been described in the literature by Lu *et al.* [20].

### 2.1. Synthesis of PLA-PEG-PLL

**Figure 1.**
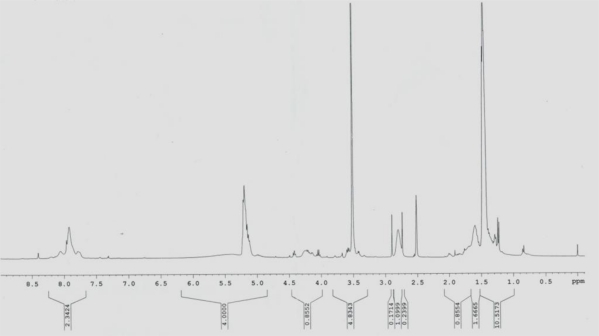
The ^1^H NMR spectra of PLA-PEG-PLL.

PLA-PEG-PLL was analyzed by ^1^H-NMR using DMSO-D_6_ (Figure 1) as a solvent. PLA: (*δ*CH_3_) = 1.56 ppm, (*δ*-CH) = 5.15 ppm; PEG: (*δ*-CH_2_) = 3.51 ppm; PLL: (*δ*(CH_2_)_3_) = 1.22–1.29, (*δ*-CH_2_) = 2.51, (*δ*(-HN-CH<)) = 4.18. The GPC of the tri-block copolymer PLA-PEG-PLL was performed to determine the molecular weight of 17,500 and the polydispersity index (PDI) of 1.17. The molecular weight of each polymer, PEG average Mn 2,000, PLA average Mn 13,500 and PLL average Mn 2,000, were also measured.

### 2.2. Formation and Physicochemical Characterization of Cationic PLA-PEG-PLL NPs and PLA-PEG-PLL NPs/DNA Complexes

**Figure 2.**
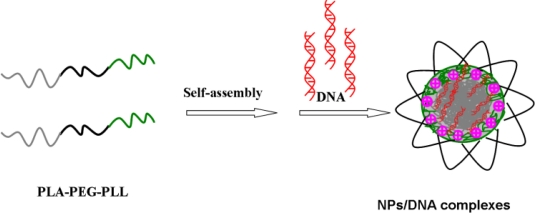
Schematic illustration of PLA-PEG-PLL NPs/DNA complexes.

In the present study, based on our previously research on PLA-PEG, we have designed and synthesized PLA-PEG-PLL which self-assembled to form cationic nanoparticles in aqueous solution, then adsorbed DNA via self-assembly nanotechnology based on alternative deposition of oppositely charged compounds. In this nanoparticle system, hydrophobic PLA segments as the core could increase the stability of the system; due to the primary ɛ-amine groups of lysine, PLL segments provide a positive charge which combine with DNA, deposited into the core; the aggregated segments are quickly stabilized by hydrophilic PEG chains which form a loop structure around the nanoparticles. The loop structure might increase the thickness of the PEG adsorption layer. The more loops at the interface, the thicker the adsorption layer [21]. PEGylation further increased the stability of the nanoparticles and reduced their interaction with proteins in the physiological fluid greatly. The schematic illustration of the preparation of NPs/DNA complexes is shown in Figure 2. This structure concord with self-assembled micelles based on PLL-PEG-PLL copolymers was described by Zhuo [22].

**Figure 3.**
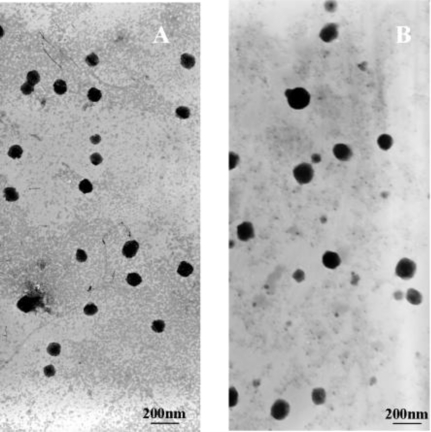
Transmission electron micrographs (TEM) of PLA-PEG-PLL NPs (**A**) and PLA-PEG-PLL NPs/DNA complexes (**B**).

The formulations of PLA-PEG-PLL NPs and PLA-PEG-PLL NPs/DNA complexes were evaluated by the particle size, polydispersity index and zeta potential. Transmission electron micrographs of the PLA-PEG-PLL NPs and NPs/DNA complexes are shown in Figure 3. It is shown that the obtained NPs exhibited a similar uniform spherical shape with a smooth surface and were separated from each other. When the N/P ratio is 15 or higher, the diameters of the complexes are all smaller than 150 nm, which is suitable for gene delivery, since it has been reported that carrier/DNA complexes with diameters larger than 150 nm were difficult for many types of mammalian cells to endocytose [23]. When the NPs/DNA ratio is 12 or lower, the diameters of complexes are relatively bigger (∼230 nm), indicating that the NPs/DNA complexes may be loose and the DNA in the complex is not thoroughly condensed. Mean particle sizes of PLA-PEG-PLL NPs and NPs/DNA complexes (N/P ratios 15:1) were 78.8 ±4.5 nm and 127.4 ±6.7 nm respectively. The polydispersity index (PDI) of two kinds of nanoparticles ranged from 0.14 to 0.18, which demonstrated a relatively narrow particle size range. Zeta potential is another important index for the stability of the NPs suspension. Analysis of the surface charge of the NP complexes showed that when the N/P ratio was increased from 9 to 15, the complexes that were initially negatively charged at approximately −10.3 mV became positively charged at +14.9 mV. Increases in the N/P ratio (up to 21) did not further modify the zeta potential value (+25 mV). A positive zeta potential of NPs/DNA complexes is necessary to ensure the uptake of nanoparticles by cells because a positive surface charge allows an electrostatic interaction between negatively charged cellular membranes and the positively charged complexes [24].

Our group previously demonstrated that the cationic surfactant CTAB modified PLA-PEG formed cationic nanoparticles, which could adsorb DNA on the surface and successfully transfer plasmid DNA into HeLa cells [16]. The cationic surfactant CTAB was shown to be effective for absorbing DNA onto the surface nanoparticles. The addition of CTAB could increase the zeta potential of the NPs in comparison to the anionic zeta potential of the non-modified NPs [17]. However, a concern with coating the surface of particles with cationic polymers is cellular toxicity. At high coating concentrations of CTAB, membrane toxicity was observed in cells [25]. Similarly, PLL has been able to change the surface charge when used as an emulsifier in the fabrication of nanoparticles [26]. The use of cationic polymers to condense DNA for transfection of cancer cells has been well studied. Recently, this interaction has been explored to enhance the gene transfection of biodegradable nanoparticles. Blum *et al.* conjugated PLL to PLGA to create an electrostatically favorable interaction between the carrier material and the DNA, and the particles were shown to successfully transfect COS cells [27]. Capan *et al.* have encapsulated DNA previously complexed with PLL into PLGA microparticles and demonstrated the ability to control release of these complexes by using different molecular weight polymers or changing the surfactant concentration [28].

### 2.3. Gel Retardation Assay

**Figure 4.**
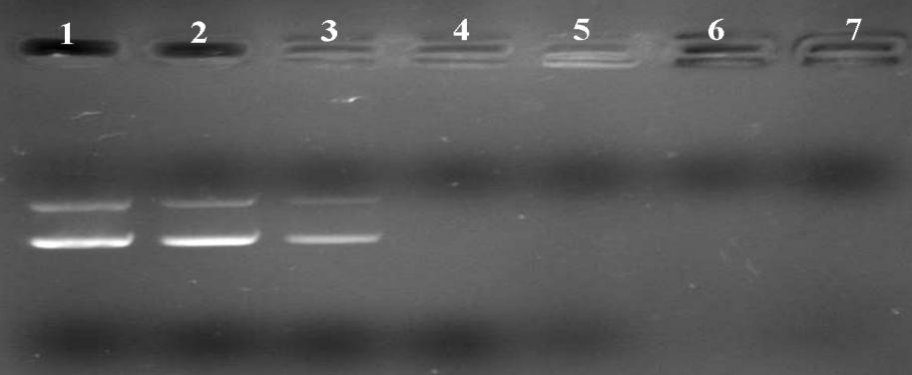
Gel retardation assay of PLA-PEG-PLL NPs/DNA complexes prepared at different N/P ratios. Lane 1: Naked DNA; Lane 2–7: The N/P ratios of PLA-PEG-PLL NPs/DNA complexes was 9:1, 12:1, 15:1, 18:1, 21:1 and 24:1, respectively.

Generally, cationic nanoparticles could efficiently adsorb anionic plasmid DNA on the surface, to form NPs/DNA complexes, primarily via electrostatic attraction. Agarose gel electrophoresis was carried out to confirm whether the plasmid DNA could be associated with the cationic PLA-PEG-PLL NPs, and to qualitatively investigate the optimal N/P ratio of NPs to DNA (defined as the molar ratio of amino groups in the PLL to phosphate groups in the DNA) for the binding efficiency. Different complexes were prepared at N/P ratios of 9:1, 12:1, 15:1, 18:1, 21:1 and 24:1 by using PLA-PEG-PLL NPs to DNA and the retardation in the migration of the plasmid DNA during agarose gel electrophoresis was examined. As shown in Figure 4, it was observed that naked plasmid DNA (lane 1) could migrate to the positive electrode under the electric field, leading to visualization of two bands corresponding to open circular and supercoiled morphology. Once DNA was associated with the NPs to form complexes, the mobility of DNA was hindered and it was detained in the well of the agarose gel. The part of DNA that did not bind the NPs migrated to the positive electrode in the same manner asa the control DNA. When the N/P ratio of PLA-PEG-PLL NPs to DNA reached 15:1 or above, almost all DNA was combined with NPs without free DNA bands visible in the lane (lane 4–7). Thus, NPs/DNA complexes prepared with a ratio of 15:1 were selected as the optimal N/P ratio of NPs to DNA for further study.

### 2.4. Heparin Replacement

**Figure 5.**
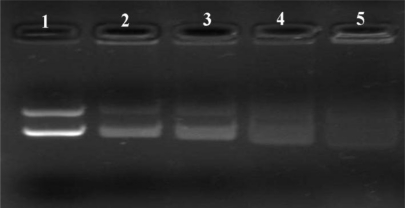
Plasmid DNA extracted from the PLA-PEG-PLL NPs/DNA complexes (N/P 15:1) by the addition of different concentration of heparin solution. Lane 1: Naked DNA; Lane2–5: PLA-PEG-PLL NPs/DNA complexes extracted with 1% heparin, 2% heparin, 5% heparin and 10% heparin, respectively.

Another essential consideration was the integrity of the DNA that had been incorporated into the NPs. The release of the plasmid DNA from the optimal N/P ratios 15:1 of PLA-PEG-PLL NPs/DNA complexes by heparin displacement with different concentration was investigated. As illustrated in Figure 5, the DNA structure still remained a complete structure corresponding to open circular and supercoiled morphology when it was extracted by heparin of final concentration 1% and 2% (lane 2 and lane 3), while with increased heparin concentration the DNA structure was degraded, especially at a final concentration of 10%, where the open circular and supercoiled morphology was not observed completely. Thus, heparin at a final concentration of 1% was selected as the optimal extraction condition for further study.

### 2.5. Stability of PLA-PEG-PLL NPs/DNA Complexes in Plasma

**Figure 6.**
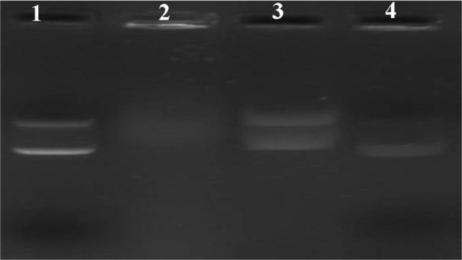
Stability of PLA-PEG-PLL NPs/DNA complexes (N/P 15:1) in 10% human plasma. Lane 1: Naked DNA; Lane 2: Naked DNA incubated with human plasma for 2 h; Lane 3: NPs/DNA complex incubated with human plasma for 2 h; Lane 4: NPs/DNA complex extracted with 1% heparin.

Agarose gel electrophoresis was carried out to investigate whether the PLA-PEG-PLL NPs/DNA complexes could be stable in human plasma. Figure 6 showed the stability with the N/P ratio 15:1 of NPs/DNA complexes in plasma. It could be observed that the plasmid DNA alone was degraded completely by nucleases in plasma with the exposure of 10% human plasma for 1 h in lane 2. However, in the presence of human plasma, NPs/DNA complexes remained relatively stable, and DNA still remained a complete structure corresponding to open circular and supercoiled morphology. This result confirmed that the PLA-PEG-PLL NPs could not only adsorb DNA efficiently onto their surfaces but could also partially protect the condensed DNA against degradation by plasma proteins. This was attributed to PEG modification, which could act to reduce the nanoparticles’ interaction with proteins in the physiological fluid and produce a shielding effect that counteracts effective DNA complexation [29].

### 2.6. DNase I Protection Assays

**Figure 7.**
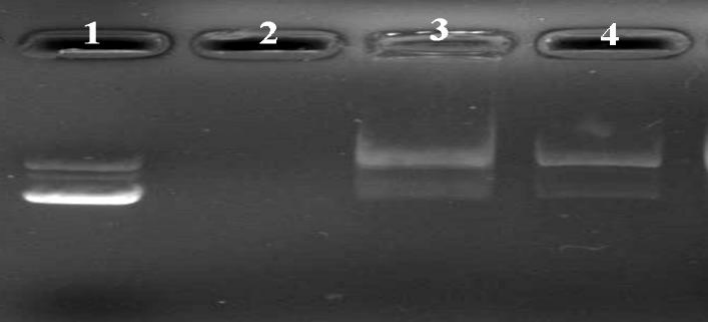
Agarose gel electrophoresis of PLA-PEG-PLL NPs/DNA complexes (N/P 15:1) after incubation with different concentrations of DNase I. Lane 1: Naked DNA; Lane 2: Naked DNA incubated with DNase I at 0.1 U/μg DNA for 30 min; Lane 3–4: NPs/DNA complexes incubated with DNase I at 0.1 and 0.2 U/μg DNA, respectively, for 30 min.

Effective condensation is a key issue for DNA stability against degradation by nucleases [30]. Deoxyribonuclease I (DNase I) or DNase I-like enzymes exist in the extracellular space [31] and cellular cytoplasm [32] and have been implicated to play a role in DNA degradation and disruption. The ability of cationic nanoparticles to protect against nuclease attack is paramount in enhancing the success of gene expression. To investigate whether PLA-PEG-PLL NPs could protect adsorbed plasmid DNA from digestion by nucleases, the complexes were exposed to DNase I at different concentrations. As illustrated in Figure 7, naked plasmid DNA (lane 2) was completely digested by DNase I at 0.1 U/μg DNA within 30 min of incubation, confirming the activity of nucleases. However, DNA extracted from the N/P ratio 15:1 of NPs/DNA complexes through heparin at final concentration of 1%, remained intact in supercoiled form in the investigated concentrations of DNase I within 30 min of incubation (lane 3–4), similar to the DNA control in lane 1. Our studies showed that PLA-PEG-PLL NPs could protect DNA against degradation by DNase I, which is one of the crucial factors for efficient gene delivery *in vitro* as well as *in vivo*.

### 2.7. Cytotoxicity of PLA-PEG-PLL NPs/DNA Complexes

**Figure 8.**
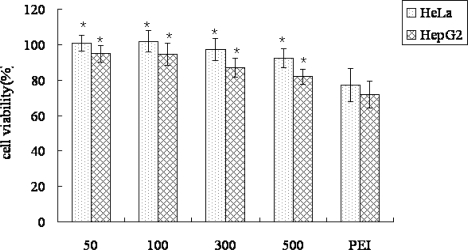
Cytotoxicity of various concentrations of PLA-PEG-PLL NPs/DNA complexes in HepG2 and HeLa cells. PLA-PEG-PLL NPs/DNA concentrations were from 50 μg/mL to 500 μg/mL (*n* = 3, *P < 0.05 compared with PEI).

Non-viral gene delivery systems have certain drawbacks in terms of cytotoxic reactions; thus evaluation of the cytotoxicity of the cationic nanoparticles is essential. The cytotoxicity of PLA-PEG-PLL NPs/DNA complexes at various concentrations was evaluated by MTT assay in HepG2 and HeLa cells, in comparison with PEI. As shown in Figure 8, PLA-PEG-PLL NPs/DNA complexes showed almost no toxicity (higher than 80%) in both HepG2 and HeLa cell lines at all the tested concentrations and the average cell viabilities of different formulations were all significantly higher than PEI/DNA (*p* < 0.05). According to these results, the PLA-PEG-PLL NPs/DNA complexes appear to be a safe carrier. The low cytotoxicity of NPs may be explained by the fact that the biodegradable tri-block copolymer PLA-PEG-PLL could electrostatically interact with plasmid DNA without any cationic surfactants.

### 2.8. *In Vitro* Transfection Studies

**Figure 9.**
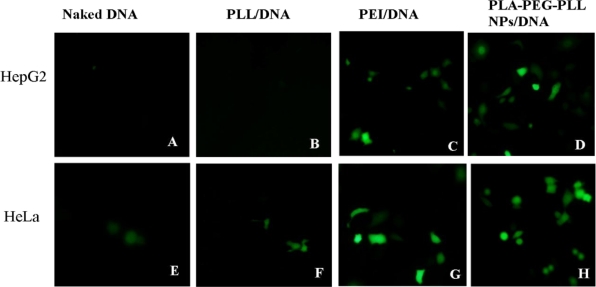
Fluorescent images of gene transfection of pEGFP-N_1_ in HepG2 (top panel) and HeLa cells (bottom) with naked DNA (**A**,**E**), PEI/DNA complexes (**B**,**F**), PEI/DNA complexes (**C**,**G**), PLA-PEG-PLL NPs/DNA complexes (**D**,**H**) (×200).

**Figure 10.**
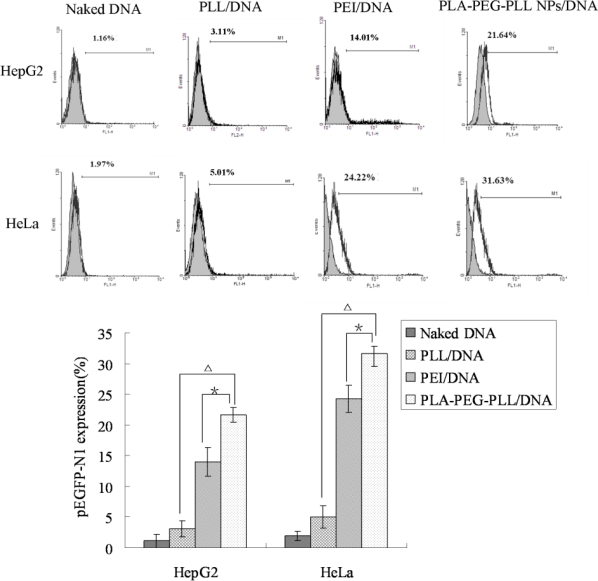
Flow cytometry of HepG2 (top panel) and HeLa cells (bottom) transfected with pEGFP-N_1_ with naked DNA, PLL/DNA, PEI/DNA and PLA-PEG-PLL NPs/DNA complexes. Gene expression was measured 24 h post transfection. The reported transfection efficiency is the ratio of the number of cells producing fluorescent signal to the number of total cells. (*P < 0.05 compared with PEI/DNA complexes, Δ*P* < 0.01 compared with PLL/DNA complexes, *n* = 3).

Designing a safe and efficient non-viral gene delivery system is essential for a variety of gene therapy applications. In the present study, all the transfection studies were performed in the presence of serum medium to verify whether the hydrophilic PEG as the loop structure could prevent absorption of plasma proteins on the surface of the nanoparticles. Transfection ability of our established vectors PLA-PEG-PLL NPs/DNA complexes was investigated in comparison with PEI/DNA complexes. From fluorescence images (Figure 9), it was shown that naked DNA and PLL/DNA complexes could barely transfect either HepG2 or HeLa cells, whereas, both PLA-PEG-PLL NPs/DNA complexes and PEI/DNA, could achieve intracellular gene tranfection. The gene transfection efficiency of the complexes was then further quantitively determined by flow cytometry and the data are shown in Figure 10.

In the preliminary tests, we chose PLL (25 kDa) as positive control. The results show that in the study of heparin replacement, no matter how high the concentration of heparin, it did not extract DNA from PLL/DNA complexes (data not shown). Furthermore, in *in vitro* transfection studies, PLL/DNA complexes barely transfected in HepG2 cells and HeLa cells, as shown in the fluorescence images, and only gene expression efficiency of 3.11% and 5.01%, respectively, was obtained (*P* < 0.01, compared with PLA-PEG-PLL NPs/DNA complexes). This might be explained by the fact that PLL condensed DNA too tightly to release DNA from the complexes, leading to the lower transfection efficiency. In the present study, PEI (25 kDa) was chosen as positive control. PEI (25 kDa) is considered as one of most efficient currently-available transfection reagents, but this molecule is not biodegradable and is relatively cytotoxicity. Compared with PEI/DNA complexes, the transfection efficiency of PLA-PEG-PLL NPs/DNA complexes still exhibited higher gene expression of up to 21.64 % and 31.63% (more than PEI/DNA at 14.01% and 24.22%) in HepG2 and HeLa cells, respectively (*P* < 0.05).

In the presence of serum, PLA-PEG-PLL NPs/DNA complexes maintained visibly higher gene transfection activity than PEI/DNA complexes. This might be explained that PEI/DNA complexes tended to adsorb anionic serum protein and form a larger aggregate, which could not get across the cell membrane and deliver the DNA to the nucleus. PLA-PEG-PLL NPs/DNA complexes with PEGylation could prevent nanoparticle aggregation and prevent the non-specific interactions with serum protein especially in the transfection medium, and accordingly improve the transfection efficiency of nanoparticles. It was also reported that PEGylation indeed increased the stability of the nanoparticles and the transfection efficiency was not affected even in the present of serum by Liu *et al.* [33]. All the results demonstrated that the PLA-PEG-PLL NPs/DNA complexes could successfully transfect cancer cells and be used as a potential delivery system for DNA in cancer gene therapy.

## 3. Experimental Section

### 3.1. Material

pEGFP-N_1_ was provided by Zhejiang University (China). PicoGreen dsDNA reagent was obtained from Molecular Probes (Invitrogen, USA). Agarose was purchased from BIO-WEST (Spain). Goldview was obtained from Beijing Saibaisheng Biological Engineering Co. (Beijing, China). DNase I enzyme was obtained from Beijing Yinfeng Century Scientific Develop Co., Ltd (Beijing, China). MTT (3-[4,5-dimehyl-2-thiazolyl]-2,5-diphenyl-2*H*-tetrazolium bromide) were purchased from Sigma-Aldrich (China). PEI (25kDa) was from Sigma-Aldrich (China). HepG2 and HeLa cell lines were obtained from American Type Culture Collection (ATCC, USA). All the other chemicals and reagents used were of analytical purity grade or higher, obtained commercially.

### 3.2. Synthesis of PLA-PEG-PLL

The tri-block copolymer PLA-PEG-PLL was synthesized by the method described previously by Lu *et al.* [20]. Briefly, tetrahydrofuran, potassium hexamethyldisilazane and ethylene oxide were mixed in ice bath for 3 days, then d,l-LA addedfor 8 h to obtain H_2_N-PEG-PLA. After acidolysis and purification, the H_2_N-PEG-PLA product (PEG 2000) was vacuum-dried at room temperature. The H_2_N-PEG-PLA was dissolved in DMF under argon atmosphere, then Lys(Z)-NCA added. The reaction mixture was stirred for 30 h at room temperature. After evaporation of the solvent, the retentate was dissolved with CH_2_Cl_2_, and precipitated with anhydrous ether and filtered. After being filtered, the product was vacuum-dried at room temperature. The product was dissolved in trifluoroacetic acid under argon atmosphere, and stirred for 4 h in an ice bath by adding 50% hydrogen bromide/acetic acid solution. Then the solution was added into HAc/MeOH mixture. The resulting copolymer PLA-PEG-PLL was obtained after being filtered.

### 3.3. Preparation of the Blank PLA-PEG-PLL Nanoparticles

The blank PLA-PEG-PLL NPs were prepared using the method previously described by Choi *et al.* [34]. Briefly, 3 mL organic polymer solution (60 mg of PLA-PEG-PLL dissolved in DMSO) was dropped at the rate of 15 mL/h in 20 mL distilled water under moderate stirring, leading to the immediate polymer precipitation. Subsequently, the solution was put in dialysis bag (molecular weight cutoff 3,500 Da, Sigma) and dialyzed against deionized water for 48 h to remove solvent. The deionized water was exchanged in 5 h intervals for 48 h. The dialyzed solution was filtered with a 0.45 μm syringe filter. Then the blank PLA-PEG-PLL NPs were collected.

### 3.4. Preparation of Gene Loaded PLA-PEG-PLL Nanoparticles

The gene loaded cationic PLA-PEG-PLL NPs to form NPs/DNA complexes were obtained by means of electrostatic attraction between the anionic plasmid DNA and the blank cationic nanoparticles. Briefly, the reporter gene encoding enhanced green fluorescence protein (pEGFP-N_1_) was mixed in different proportions with the cationic PLA-PEG-PLL NPs, which was suspended in NaCl (150 mM), under gentle vortexing for 20 s and incubated for 30 min at room temperature to facilitate complexation. The resultant PLA-PEG-PL NPs/DNA complexes were directly used for further study.

### 3.5. Agarose Gel Electrophoresis of NPs/DNA Complexes

In order to further confirm the adsorption of DNA onto the surface of cationic PLA-PEG-PLL NPs, agarose gel electrophoresis was carried out. The gels were prepared with 0.8% (w/v) agarose in 20 mL TAE buffer (40 mM Tris, 40 mM Acetic acid, 1 mM EDTA, pH 8.5) containing 2 μL goldview as stain. A fixed amount (0.5 μg) of DNA was incubated with various amounts of PLA-PEG-PLL NPs in NaCl solution (the N/P ratio of PLA-PEG-PLL and DNA was 9:1, 12:1, 15:1, 18:1, 21:1 and 24:1 respectively). The resultant PLA-PEG-PLL NPs/DNA complexes and control plasmid DNA were applied to gel electrophoresis at a constant 90 V for 20 min. After the electrophoresis, images were obtained using UV transilluminator and Multimage ^TM^ Light Cabinet (Alpha Imagers EC, Alpha Innotech Corporation) to show the location of DNA.

### 3.6. Morphology, Particle Size and Zeta Potential

The morphology of the PLA-PEG-PLL NPs and PLA-PEG-PLL NPs/DNA complexes at N/P ratio 15:1 was examined by transmission electronic microscopy (TEM) (JEM-1200EX, Japan). Samples were prepared by placing a drop of nanoparticle suspension onto a copper grid and air-dried, following negative staining with one drop of 2% aqueous solution of sodium phosphotungstate for contrast enhancement. The air-dried samples were then directly examined under the transmission electron microscope.

The mean particle size and zeta potential of the nanoparticles were analyzed by photon correlation spectroscopy (PCS) with a Zetasizer 3000 (Malvern Instruments, Malvern, England). All measurements were carried out in triplicate. The average particle size was expressed in volume mean diameter and the reported value was represented as mean ±S.D. (*n* = 3).

### 3.7. Integrity Analysis of DNA

To investigate the integrity of DNA from the cationic PLA-PEG-PLL NPs, the plasmid DNA by heparin displacement was studied. In brief, PLA-PEG-PLL NPs/DNA complexes were made at N/P ratios of 15:1 of NPs/DNA, after 30 min of incubation, different final concentration of heparin solution (1%, 2%, 5%, 10%) was added and the samples were incubated in shaking water bath (100 rpm) for 3 h at 37 °C. The configuration of plasmid DNA in the complexes after heparin extraction was analyzed by gel electrophoresis with untreated naked DNA as a reference. The samples were applied to a 0.8% (w/v) agarose gel in TAE buffer as described above.

### 3.8. Stability Study in Human Plasma

The stability of the PLA-PEG-PLL NPs/DNA complexes was determined in the presence of 20% human plasma at 37 °C [35]. 10 μL of NPs/DNA complexes (N/P 15:1) was incubated with 10 μL of human plasma at 37 °C for 2 h, and naked DNA incubated with 10 μL of human plasma was used as a control. To asses the integrity of DNA loaded in the nanoparticles, it was dissociated from the cationic nanoparticles by adding heparin solution at final concentration of 1% (w/v) and the suspension was then incubated in a shaking water bath (100 rpm) for 3 h at 37 °C. The configuration of plasmid DNA was analyzed by gel electrophoresis and untreated naked DNA was used as a reference.

### 3.9. DNase I Protection Study

To test whether PLA-PEG-PLL NPs/DNA complexes can protect the loaded plasmid DNA from nucleases digestion, the results of DNase I mediated digestion was evaluated using agarose gel electrophoresis. Cationic nanoparticles were prepared at N/P ratio of 15:1 of NPs/DNA, incubated with different amounts of DNase I (0.1 and 0.2 U/μg DNA) in DNase I/Mg^2+^ digestion buffer (50 mM, Tris-HCl, pH 7.6, and 10 mM MgCl_2_). Naked DNA (1 μg) was treated with DNase I at 0.1 U/μg DNA as a reference. Then the samples were incubated at 37 °C for 30 min. 5 μL EDTA solution (0.5 M, pH 8.0) was added to inactivate the DNase I. The integrity of DNA loaded in the nanoparticles was studied by adding heparin solution at a final concentration of 1% (w/v) [36]. Then the PLA-PEG-PLL NPs/DNA complexes containing heparin were placed in a 37 °C shaking water bath at 100 rpm for 3 h. After that, the suspension was analyzed by gel electrophoresis as described above. Untreated naked DNA was applied to the gel as a control.

### 3.10. *In Vitro* Cell Viability

Cytotoxicity of PLA-PEG-PLL NPs/DNA complexes was assayed by MTT assay in HepG2 and HeLa cell lines [37]. The cells were seeded into a 96-well microtiter plates at a density of 8 × 10^3^ cells per well in 0.2 mL of RPMI 1640 supplemented with 10% fetal bovine serum (FBS) and antibiotics in 5% CO_2_ incubator at 37 °C. After 24 h, the culture medium was replaced by 200 μL fresh serum-free RPMI 1640 medium with different concentrations of the nanoparticles (expressed as PLA-PEG-PLL concentration, 50, 100, 300, 500 μg/mL) and PEI (25 kDa) in comparison. The PEI/DNA complexes were formed at a molar ratio of PEI nitrogen to DNA phosphate (N/P ratios) of 10:1 [38]. The applied dosage of PEI/DNA complexes was proportional to that used in transfection experiments. After incubating for 24 h, the effect of different treatments on cell viability was assessed by the MTT assay. Typically, 5 mg/mL of MTT in PBS were added to each well reaching a final concentration of 0.5 mg/mL MTT and incubated for 4 h. Then the supernatants were removed and the formazan crystals were dissolved in 100 μL DMSO. Aliquots were drawn from each well and the absorbance at 570 nm was determined by a microplate reader (Model 680, BIO-RAD, USA). Cells without addition of MTT were used as blank to calibrate the spectrophotometer to zero absorbance. The relative cell viability (%) compared to control cells were calculated by (*A*_sample_/*A*_control_) × 100. All treatments were done in quadruplicates and all experiments were repeated in triplicates. A paired t-test with *P* < 0.05 was used to establish statistically significant differences between treatments.

### 3.11. *In Vitro* Transfection Efficiency

The transfection activity of PLA-PEG-PLL NPs/DNA complexes was evaluated in HepG2 and HeLa cell lines. The cells were seeded into 12-well plates at a density of about 3 × 10^5^ cells per well in 1 mL of RPMI 1640 culture medium with 10% FBS, 24 h prior to transfection. At a confluence level of 70–80%, cells were washed twice with PBS, and, respectively, incubated with 500 μL of media (with or without 10% FBS) containing 1 μg DNA in transfection vectors at 37 °C, PLL/DNA complexes and PEI/DNA complexes were used as positive control, 1 μg naked DNA was used as negative control. The cells were incubated with the vectors for 4 h, in the presence of serum medium. The transfection media was then replaced with 1 mL of fresh complete culture media, and the cells were incubated sequentially until 24 h post transfection. Detection of expression of pEGFP-N_1_ was carried out using an inverted fluorescent microscope with an attachment for fluorescent observation (OLYMPUS, ZX71, Japan) and the picture was captured using a 4009 objective. After that, all cells were harvested for trypsinization and washed in PBS for three times and cell-associated fluorescence was determined by a FACSCalibur flow cytometer (BD Biosciences) for quantitative study. For each sample, 10,000 events were collected and fluorescence was detected. Signals were amplified in logarithmic mode for fluorescence to determine the positive events by a standard gating technique. The percentage of positive events was calculated as the events within the gate divided by the total number of events, excluding cell debris. Transfection experiments were performed in triplicate [39].

## 4. Conclusions

Development of safe and efficient non-viral vectors receives tremendous attention in the hope to find a substitute for viral vectors. The biodegradable tri-block copolymer PLA-PEG-PLL was synthesized by the ring-opening polymerization of Lys(Z)-NCA onto amine-terminated NH_2_-PEG-PLA, then acidolysis to remove benzyloxycarbonyl. The novel cationic PLA-PEG-PLL NPs/DNA complexes could successfully transfer pEGFP-N_1_ into HepG2 and HeLa cells, and produced higher transfection efficiency than PEI, while showing lower cytotoxicity than PEI. The results of this study suggest that the tri-block copolymer PLA-PEG-PLL in this work could offer a novel promising non-viral nano-device for gene therapy. Our future study will focus on the surface-modification of the biodegradable tri-block copolymer PLA-PEG-PLL with various kinds of ligands to achieve active targeting to tissues and cells to build the “perfect vector” for systemic gene therapy against cancer.
